# SETD1A Mediated H3K4 Methylation and Its Role in Neurodevelopmental and Neuropsychiatric Disorders

**DOI:** 10.3389/fnmol.2021.772000

**Published:** 2021-11-03

**Authors:** Shan Wang, Anna Bleeck, Nael Nadif Kasri, Tjitske Kleefstra, Jon-Ruben van Rhijn, Dirk Schubert

**Affiliations:** ^1^Department of Cognitive Neuroscience, Donders Institute for Brain, Cognition and Behaviour, Radboudumc, Nijmegen, Netherlands; ^2^Department of Human Genetics, Radboudumc, Nijmegen, Netherlands; ^3^Centre of Excellence for Neuropsychiatry, Vincent van Gogh Institute for Psychiatry, Venray, Netherlands

**Keywords:** SETD1A, neurodevelopmental disorders (NDD), psychiatric disorders, chromatin modification, histone methlyation, schizophrenia

## Abstract

Posttranslational modification of histones and related gene regulation are shown to be affected in an increasing number of neurological disorders. SETD1A is a chromatin remodeler that influences gene expression through the modulation of mono- di- and trimethylation marks on Histone-H3-Lysine-4 (H3K4me1/2/3). H3K4 methylation is predominantly described to result in transcriptional activation, with its mono- di- and trimethylated forms differentially enriched at promoters or enhancers. Recently, dominant mostly *de novo* variants in *SETD1A* have clinically been linked to developmental delay, intellectual disability (DD/ID), and schizophrenia (SCZ). Affected individuals often display both developmental and neuropsychiatric abnormalities. The primary diagnoses are mainly dependent on the age at which the individual is assessed. Investigations in mouse models of SETD1A dysfunction have been able to recapitulate key behavioral features associated with ID and SCZ. Furthermore, functional investigations suggest disrupted synaptic and neuronal network function in these mouse models. In this review, we provide an overview of pre-clinical studies on the role of SETD1A in neuronal development. A better understanding of the pathobiology underlying these disorders may provide novel opportunities for therapeutic intervention. As such, we will discuss possible strategies to move forward in elucidating the genotype-phenotype correlation in *SETD1A* associated disorders.

## Introduction

Chromatin modification and the related regulation of gene expression patterns have been linked to several neurological disorders, in particular neurodevelopmental (NDD) or neuropsychiatric disorders (NPD; Gabriele et al., 2018; Satterstrom et al., 2020; Mossink et al., 2021). The basic building block for chromatin is the nucleosome, which consists of a 147 base pair DNA structure that wraps around an octamer of the four core histones H3, H4, H2A, and H2B. Chromatin structure dynamics are closely associated with DNA accessibility and the efficiency of DNA transcription and replication. Currently, it is well-recognized that epigenetic mechanisms such as post-translational modification of histones can control chromatin structure and organization, thereby influencing gene expression (Mossink et al., 2021).

One such posttranslational chromatin modification is the methylation of lysine groups at histones through different enzymes. Here we focus on mono-, di- and trimethylation of lysine 4 at histone H3 (H3K4me1/2/3). H3K4 methylation is generally implicated in transcription (Kusch, 2012). H3K4me1, 2, and 3 localizes to specific parts of the nucleosome: H3K4me1 is distributed at enhancer regions, H3K4me2 is found in nucleosomes further downstream in the body of genes, and H3K4me3 is located in nucleosomes near the transcription start sites (TSS) of expressed genes, presumably at promoter regions (Kusch, 2012). Over the past decades, enzymes of the type 2 lysine methyltransferase (KMT2, also known as mixed lineage leukemia; MLL) family have been found responsible for bulk H3K4 methylation. This is a highly conserved family, composed of six members. These six genes all contain a Su(var)3–9, Enhancer-of-zeste and Trithorax (SET) and post-SET domain, which are together responsible for the proteins’ methyltransferase activity and enable regulation of important aspects of cell physiology and development (Crump and Milne, 2019).

In this review, we will focus on SETD1A (also known as KMT2F), the main mammalian H3K4me1/2/3 methyltransferase. Current research associates SETD1A dysfunction with neurodevelopmental disorders (NDDs), early onset epilepsy, and schizophrenia (SCZ; Singh et al., 2016; Yu et al., 2019; Kummeling et al., 2020). This suggests that SETD1A plays a crucial role both during brain development as well as in maintaining healthy brain function.

## The Function of SETD1A Is Highly Conserved Throughout Evolution

The methyltransferase activity of SETD1A is dependent on its interactions with several other proteins, which form a highly conserved complex, designated the “complex of proteins associated with Set1” (COMPASS). Originally identified in yeast (Ruthenburg et al., 2007), COMPASS complexes remain rather conserved during evolution: in *Drosophila* there are three Set1-like H3K4 methyltransferase complexes with three different enzymatic subunits: Set1, Trithorax (Trx), and Trithorax-related (Trr), whereas mammals have six Set1-like H3K4 methyltransferases: SETD1A/B and MLL1–4. Based on the sequence homology of the SET-containing enzymatic subunits and composition of the COMPASS, it was defined that MLL1/2 are homologous to Trx, MLL3/4 are homologous to Trr, and SETD1A/B are homologous to dSet1 (Mohan et al., 2011). The conserved structure of COMPASS complexes is also reflected in the function of the proteins, with Set1 in *Drosophila* and SETD1A/B in mammals both being considered as the major H3K4 trimethyl transferases (Ardehali et al., 2011; Clouaire et al., 2012). This underscores the essential biological function of SETD1A.

## SETD1A Regulates Gene Transcription as Part of A Multi-Subunit Protein Complex

**Figure 1:**
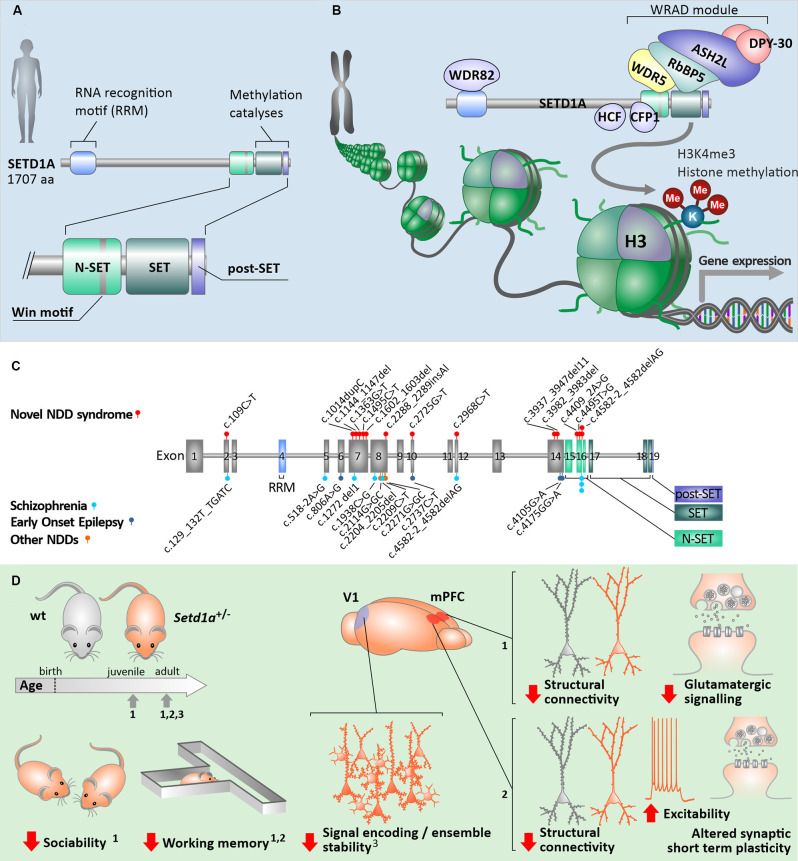
Human SETD1A in health and neurological disorders. **(A)** General build-up of the human SETD1A protein and its key binding/catalytic motifs. **(B)** Principle of H3K4me3 histone modification *via* the COMPASS protein complex with SETD1A. **(C)** Known mutations in the coding sequence of *SETD1A* and associated neurological disorders [data from Singh et al. (2016); Yu et al. (2019); Kummeling et al. (2020)]. **(D)** Consequences of *Setd1a* haploinsufficiency in *Setd1a*^+/–^ adult mouse models on behavior as well as neuronal (network) structure and function, compared to wildtype animals (wt). The relevant studies were performed at different developmental timepoints, each of which indicated by arrows on the developmental timeline. Numbers indicate the referenced sources: data from Nagahama et al. (2020) (1); (Mukai et al., 2019) (2); (Hamm et al., 2020) (3); V1—primary visual cortex, mPFC—medial prefrontal cortex.

In humans, SETD1A contains highly conserved SET and post-SET domains at the C terminus, like all other members of the KMT2 family (Figure 1A). Additionally, adjacent to the SET domain in the N-terminal direction, the n-SET domain (including the conserved WDR5 binding “WIN” motif) plays an important role in H2B ubiquitylation and eventually downstream H3K4 methylation (Kim et al., 2013). Near the N-terminal region, SETD1A also contains an RNA recognition motif (RRM) domain. All mammalian SET1-family complexes constitute WDR5, RBBP5, ASH2, and DPY30 forming the four subunit sub-complex WRAD, which is essential for H3K4 methyltransferase activity (Ernst and Vakoc, 2012; Figure 1B). Whereas WRAD is an essential sub-complex for members of the SET1-family in general, the functional SETD1A complex requires additional subunits. Such additional protein-protein complexes are formed with CFP1, WDR82, and HCF1 (Figure 1B). CFP1, also known as CXXC1, serves as the predominant targeting module for the SETD1A complex and plays a key role in guiding H3K4me3 deposition and proper expression of target genes (Brown et al., 2017). WDR82 interacts with SETD1A *via* the RRM domain. It mediates binding to the Ser5-phosphorylated C-terminal domain of RNA polymerase II, which results in the initiation of transcription by recruiting the SETD1A complex to transcription start sites (Lee and Skalnik, 2008). Lastly, HCF1 interacts with SETD1A through the HCF-1-binding motif (HBM). Through this interaction, the SETD1A complex is recruited to E2F-responsive promoters, where it can induce histone methylation and transcriptional activation and is involved in the regulation of cell cycle-related mechanisms (Tyagi et al., 2007). Taken together, this suggests that SETD1A can perform a multitude of biological functions, depending on specific interactions between subunits within the complex.

## SETD1A Dysfunction in Neurodevelopmental and Neuropsychiatric Disorders

All six human KMT2 family complexes are widely expressed in different tissues and exhibit non-redundant cellular functions (Vallianatos and Iwase, 2015). This may explain why mutations in each of the different *KMT2* family members have been shown to be causally linked to different syndromes and disorders (Vallianatos and Iwase, 2015). For example, mutations in *KMT2A* (*MLL1*) are associated with Wiedemann-Steiner syndrome, *KMT2C* (*MLL3*) with Kleefstra syndrome spectrum (Kleefstra et al., 2012; Frega et al., 2020), whereas *KMT2D* (*MLL4*, in humans, also called *MLL2*) mutations lead to Kabuki Syndrome 1 (Min Ko et al., 2017; Sobreira et al., 2017; Sun et al., 2017). In several studies with the focus on *de novo* mutations in humans, loss of function (LoF) of *SETD1A* was identified as a high-risk contribution to the etiology of SCZ (Takata et al., 2014; Singh et al., 2016; Birnbaum and Weinberger, 2017). Initially, this led to the proposition that reduced SETD1A function is a monogenic cause of SCZ. More recently this conceptual linking has been extended by associating mutations in *SETD1A* with disorders that are clinically characterized as NDDs, symptomatically represented with developmental delay, intellectual disability, behavioral problems as well as early onset epilepsy (Yu et al., 2019; Kummeling et al., 2020). Thus different individuals with SETD1A deficiency can show hallmarks of either impaired brain development and/or SCZ (Kummeling et al., 2020).

So far, the majority of individuals with a *SETD1A* mutation that report developmental problems were recruited at pediatric ages, whilst individuals reported with variants associated with SCZ were recruited at adolescent/adult ages. Specific symptoms for individuals diagnosed with SCZ typically show a later onset during the lifetime and are composed of a variation of delusions and hallucinations in addition to cognitive impairment and a decrease in social skills (Owen et al., 2016). Moreover, SETD1A-deficient individuals diagnosed with developmental problems have increasing symptoms typically associated with neuropsychiatry, including aggressive behaviors and anxiety, whereas some individuals diagnosed with SCZ also exhibited learning difficulties, which are more typically regarded as developmental problems (Owen et al., 2016; Singh et al., 2016). Therefore it is likely that *SETD1A* deficiency is associated with a variable longitudinal course and phenotypic spectrum across the lifespan. Taken together, LoF of *SETD1A* may lead to a neurodevelopmental syndrome that includes neuropsychiatric phenotypes typical for SCZ (Singh et al., 2016; Kummeling et al., 2020) and some individuals diagnosed with an NDD at pediatric ages might develop SCZ later in life.

Heterozygous mutations in the *SETD1A* gene, which is located on chromosome 16p11.2, have been shown to occur in multiple exons along the gene, predominantly 5’ but not within the catalytic SET domain. These mutations are suggested to result in LoF of the domain responsible for the methylation activity of the protein (Figure 1C; Table 1). Other mutations, located more upstream in the gene, are mostly frameshift mutations predicted to lead to a premature stop codon and reduced *SETD1A* expression, without a dominant negative function induced by the mutant allele (Cameron et al., 2019). Most of the known mutations are *de novo* (Table 1), and there are two variants (c.2968C>T; c.4582–2delAG) that have been reported in both developmental disorders and SCZ (Singh et al., 2016; Kummeling et al., 2020). Currently, there is no convincing evidence for the specific type of mutation nor the exon loci of the various *SETD1A* mutations to be reliable predictors for the clinical consequences of the affected individual (Singh et al., 2016; Yu et al., 2019; Kummeling et al., 2020). Insight into the specific molecular and cellular (neuronal) mechanisms affected by LoF of *SETD1A* will shed more light on how neurological disorders might be established in the brain.

**Table 1:** SETD1A species-specific properties and mutation locations in the Human *SETD1A* gene.

Species-specific properties of SETD1A				
	Human	Mouse	*Drosophila*	Yeast
Gene	SETD1A, KMT2F, KIAA0339	Setd1a, KMT2F, mKIAA0339	Set1	SET1, KMT2
Chromosome	16p11.2	7F3	3L	VIII
Ensemble ID	ENSG00000099381	ENSMUSG00000042308	FBgn0040022	YHR119W
Transcript length (bps)	5,991	6,487	5,495	3,243
Exons	19	19	6	1
Protein length (amino acids)	1,707	1,716	1,641	1,080
Protein size (kDa)	186	186	188	123
**Mutation locations described for human *SETD1A***				
**Exon location**	**Mutation**	**Type**	**Associated phenotype**	
**2**	c.129_132T>TGATC	frameshift	SCZ^1^	
**2**	c.109C>T	nonsense	NDD^2^	
**5**	c.518-2A>G	splice acceptor	SCZ^1^	
**6**	c.806A>G	missense	Epilepsy^3^	
**7**	c.1272del1	frameshift	SCZ^1^	
**7**	c.1014dupC	frameshift	NDD^2^	
**7**	c.1144_1147del	frameshift	NDD^2^	
**7**	c.1363G>T	nonsense	NDD^2^	
**7**	c.1495C>T	nonsense	NDD^2^	
**7**	c.1602_1603del	frameshift	NDD^2^	
**8**	c.1938C>G	stop gained	SCZ^1^	
**8**	c.2114G>GC	frameshift	NDD^1^	
**8**	c.2204_2205del	frameshift	SCZ^1^	
**8**	c.2288_2289insA	frameshift	NDD^2^	
**8**	c.2209C>T	stop gained	NDD^1^	
**8**	c.2271G>GC	frameshift	NDD^1^	
**10**	c.2725G>T	nonsense	NDD^2^	
**10**	c.2737C>T	missense	Epilepsy^3^	
**12**	c.2968C>T	stop gained	SCZ^1^, NDD^1,2^	
**14**	c.3937_3947del11	frameshift	NDD^2^	
**14**	c.3982_3983del	frameshift	NDD^2^	
**14**	c.4105G>A	missense	Epilepsy^3^	
**14**	c.4175G>A	missense	Epilepsy^3^	
**15**	c.4409-2A>G	splice acceptor	NDD^2^	
**15**	c.4495T>G	missense	NDD^2^	
**16**	c.4582-2delAG>-	splice acceptor	SCZ^1^, NDD^1,2^	

*^1.^(Singh et al., 2016); ^2.^(Kummeling et al., 2020); ^3.^(Yu et al., 2019)*.

## Role of SETD1A in Neurodevelopment—Lessons from Rodent Models

Studying mouse models for SETD1A deficiency has recently provided important information about the etiology of SETD1A associated neurological disorders. Recent studies focused on two different genetic mouse models for *Setd1a* haploinsufficiency. While one model mimics a human *de novo* frameshift mutation in exon 7 (Nagahama et al., 2020), a second model contains a LacZ/Neo cassette upstream of exon 4 that leads to LoF of *Setd1a* (Mukai et al., 2019; Hamm et al., 2020). Whereas homozygous mutations in *Setd1a* are lethal (Bledau et al., 2014), *Setd1a*^+/–^ mice of both models are viable and display distinct behavioral phenotypes (Figure 1D). *Setd1a*^+/–^ mice express impairments in social behavior as well as in working memory and learning (Mukai et al., 2019; Nagahama et al., 2020) which resemble some of the known deficits in human individuals with *SETD1A* haploinsufficiency, i.e., reductions in social skills, learning difficulties and cognitive impairments (Owen et al., 2016; Singh et al., 2016; Kummeling et al., 2020). Interestingly, *Setd1a*^+/–^ mice with an LoF in exon 7 also show hyperactivity, deficiency in novel object recognition, and impaired avoidance from aversive situations (Nagahama et al., 2020). Such behavioral phenotypes have not been reported for *Setd1a*^+/–^ mice with an LoF in exon 4 (Mukai et al., 2019; Hamm et al., 2020). It remains unresolved whether these differences in behavior are related to the exon loci of the mutation, the animal strains, or to differences in the study design.

Overall, even though differences between mouse models may exist, in rodents *Setd1a* haploinsufficiency seems to result in alterations of working memory and learning, as well as deficits in sociality. These consequences of SETD1A deficiency seem to span across evolution, as deficits in associative learning have been reported in *Drosophila* with a loss of the *SETD1A* homolog Set1 as well (Kummeling et al., 2020).

## Neuronal Phenotypes of SETD1A Dysfunction

Although it is evident that *SETD1A* mutations cause biological vulnerability to a broad neurodevelopmental phenotypic spectrum, the underlying cellular and molecular mechanisms remain poorly understood. Nevertheless, *Setd1a*^+/–^ rodent models have already revealed information about the function of SETD1A during neuronal development and gave us the first insight into how *Setd1a* mutations may result in observed behavioral phenotypes.

SETD1A cooperates with Histone Cell Cycle Regulator (HIRA), an epigenetic regulator involved in neurogenesis (Li and Jiao, 2017). Together, these two regulators increase H3K4me3, leading to an increase in β-catenin, which is a key component of the canonical Wnt/β-catenin pathway. Wnt/β-catenin signaling plays an important role in promoting neural stem cell proliferation whereas inhibiting differentiation into mature neurons (Zhang et al., 2011). Concordantly, deletion of *Setd1a* severely affects the proliferation of not only embryonic stem cells (ESCs) but also epiblast stem cells, neuronal stem cells, and induced pluripotent stem cells (iPSCs), possibly by influencing the cell cycle distribution through an increased G1 phase time but a reduced S phase (Bledau et al., 2014). Additionally, ESCs with *Setd1a* deletion fail to differentiate entirely, suggesting severe differentiation deficits (Bledau et al., 2014). This indicates that SETD1A deficiency is associated with a disturbed balance between proliferation and differentiation, possibly through Wnt/β-catenin signaling. This balance is important for early cortical development and defects herein are thought to be an underlying cause for developmental disorders (Ernst, 2016). Taken together, altered neuronal proliferation and differentiation could contribute to the NDD phenotypes found in individuals with *SETD1A* haploinsufficiency. The understanding of how and to which extent SETD1A may also influence neuronal migration remains limited. However, recently it was shown that by introducing a missense mutation p.R913C on exon 10 of *SETD1A* into E14.5 embryonic mouse brain, neurons migrated faster to the superficial cortical layers when assessed at birth (Yu et al., 2019). This suggests that this specific mutation may disturb the normal process of cortical development and may lead to long-term consequences for the formation of neuronal circuits. Further studies focusing on other mutation types such as frameshift mutations or other mutation loci are needed to better understand the role of SETD1A in neuronal migration.

Deficits in neurogenesis and potentially altered neuronal migration associated with SETD1A deficiency raise questions about the consequences for neuronal network organization and communication. In the prefrontal cortex of mice, *Setd1a* mRNA can be detected at various developmental stages, from E14.5 until 4 months postnatally (Mukai et al., 2019; Yu et al., 2019). SETD1A protein predominantly expresses in neurons, not glial cells, and such SETD1A-positive neurons are distributed over all cortical layers except L1 (Mukai et al., 2019). Synaptogenesis is a key developmental process for neuronal circuitry formation in both humans and rodents, with the critical period of synaptogenesis occurring during the first 3 postnatal weeks in rodents (Semple et al., 2013). Therefore disruption in SETD1A function in particular within this time window is supposed to result in abnormal neuronal network organization. Indeed, *Setd1a*^+/–^ mice show reduced neuronal connectivity by means of reduced spine density, particularly mushroom spine density on pyramidal neurons (Mukai et al., 2019; Nagahama et al., 2020; Figure 1D). Dysregulated spine density is known to be associated with multiple neurological disorders, such as Down syndrome and SCZ (Geschwind and Levitt, 2007; Nishiyama, 2019; Lo and Lai, 2020). Furthermore, reduced axonal projections patterns of cortical neurons from *Setd1a*^+/–^ mice have been suggested to result in changes in axonal connectivity (Mukai et al., 2019). Taken together, these data implicate that *Setd1a* deficiency during rodent brain development results in deficits in general neuronal circuitry formation.

The structural neuronal circuitry abnormalities observed in *Setd1a*^+/–^ mouse models have also been associated with altered neuronal communication. On the one hand, *Setd1a* haploinsufficiency could be related to enhanced intrinsic neuronal excitability (Mukai et al., 2019), on the other hand, there is accumulating evidence for disturbed synaptic function in adolescent as well as adult *Setd1a*^+/–^ mice **(Figure 1D)**. Whereas cortical L2/3 pyramidal neurons of *Setd1a*^+/–^ mice have been found to receive normal inhibitory inputs from spontaneously active networks, in such neurons the excitatory drive appears to be significantly reduced (Nagahama et al., 2020). This reduction seems to be elicited by changes in both the number of functional excitatory synapses present as well as the strength of the excitatory inputs. It is to be expected that altered neuronal excitability and impaired excitatory glutamatergic communication result in altered neuronal signal processing. Indeed, aberrant ensemble activity and oscillations in the primary visual cortex of *Setd1a*^+/–^ mice were previously identified, which could contribute to disruptions in sensory processing circuits (Hamm et al., 2020). This finding may offer circuit-level evidence for sensory-processing dysfunctions observed in neuropsychiatric disorders.

Furthermore, in mature cortical circuitries of *Setd1a*^+/–^ mice, changes in excitatory synaptic short-term plasticity have been observed, such as an increase in short-term depression (Mukai et al., 2019). There is currently no evidence for altered long-term plasticity in *Setd1a*^+/–^ mice. However, functional data indicates *Setd1a^+/–^* mice show alterations in their NMDAR subunit composition, which are key contributors to NMDAR-dependent long-term potentiation of synaptic signaling (Nagahama et al., 2020). Since synaptic plasticity, including short-term plasticity, is regarded as the cellular basis for learning and memory (Elgersma and Silva, 1999; Jaaskelainen et al., 2011), altered signal processing in combination with altered synaptic plasticity as observed in mature neuronal networks of *Setd1a*^+/–^ mice may be a contributing factor to the learning deficits observed from patients with *SETD1A* mutation.

Several molecular mechanisms may lead to structural and functional neuronal circuitry alterations in *Setd1a*^+/–^ mice. SETD1A, as an epigenetic regulator, is involved in the regulation of downstream gene expression. In cross-species investigations, it has been shown that *Setd1a* deficiency leads to significant changes in the transcriptomic profile of rodents (Mukai et al., 2019; Yu et al., 2019; Nagahama et al., 2020), and humans (Cameron et al., 2019). Both upregulation and downregulation of SETD1A target genes have been observed. Targets of SETD1A are highly expressed in pyramidal neurons, and dysregulated genes caused by *Setd1a* deficiency are enriched in annotations associated with synaptic functions such as “synapse organization” and “chemical synaptic transmission” (Mukai et al., 2019). Several genes important for the formation of excitatory synapses, such as Homer1, PTPRO, and ABI1, are downregulated in the mPFC of *Setd1a*^+/–^ mice (Nagahama et al., 2020), whereas SLITRK4, which is important for neurite outgrowth and excitatory synapse formation, has been found to be upregulated (Mukai et al., 2019). This upregulation of SLITRK4 may contribute to the observed alterations in neuronal morphology and spine densities in *Setd1a*^+/–^ mice.

Thus, current research suggests a crucial role of SETD1A in the development and maintenance of neuronal network function. These insights from rodent models with *SETD1A* haploinsufficiency may shed light on the question of how SETD1A deficiency may result in neurophysiological and clinical phenotypes.

## An Outlook Towards Human Models for Neuropsychiatric Disorders

Current data provide strong evidence for reduced SETD1A expression being causative for neurodevelopmental and neuropsychiatric disorders in humans. However, the biological mechanisms affected by SETD1A and histone methylation have only recently been unraveled and current knowledge has been limited to animal models. These models can provide essential and valuable insight into general mechanisms that may underlie disease and they also allow testing behavioral interventions. However, they still lack translational power. This is especially relevant in the context of epigenetic regulation as well as neuropsychiatric disorders such as SCZ, which often show highly human-specific phenotypes. Therefore, research in a human neuronal context could eventually provide deeper insight into the molecular processes affected by SETD1A deficiency.

Human iPSCs are a promising tool that revolutionized the way of human disease modeling. iPSCs have been applied to the study of a large number of diseases and pioneered the concept of “disease in a dish” (Shi et al., 2017). Human neuronal networks derived from patient iPSCs or from iPSCs with introduced disease-related mutations allow investigations in human genetic background. Currently, there are no published data on patients or genetically modified iPSC models with SETD1A deficiency. However, functional and molecular phenotyping of SETD1A deficient human iPSCs-derived neuronal networks could further elucidate how SETD1A affects gene expression associated with particular aspects of neuronal function and network maturation in human neuronal circuitry. Furthermore, such a platform can be used to investigate novel agents (Sohal and Rubenstein, 2019) that can enhance the function of SETD1A, which are of interest for the development of novel therapeutic interventions. One of the main challenges in the field of drug development is that we lack a complete understanding of the way epigenetic modification of histone marks can modulate neuronal function (Berger, 2007). For example, there is crosstalk between H3K9me and H3K4me (Li et al., 2008; Matsumura et al., 2015), which indicates that chemicals regulating H3K9me can possibly also influence H3K4me. Drug interventions in *Setd1a*^+/–^ rodent models showed that treatment with the demethylation inhibitor ORY1001 in adult mice could rescue cognitive and circuitry deficits (Mukai et al., 2019). This indicates that pharmacological interventions may hold therapeutic potential also on the established and matured brain. Further investigation is however required to better characterize the potential of ORY1001 for clinical use. Here human neuronal models may be the most promising avenue regarding the development of novel therapeutic interventions.

## Author Contributions

SW, AB, TK, NN, J-RVR, and DS designed and wrote the review. All authors contributed to the article and approved the submitted version.

## Conflict of Interest

The authors declare that the research was conducted in the absence of any commercial or financial relationships that could be construed as a potential conflict of interest.

## Publisher’s Note

All claims expressed in this article are solely those of the authors and do not necessarily represent those of their affiliated organizations, or those of the publisher, the editors and the reviewers. Any product that may be evaluated in this article, or claim that may be made by its manufacturer, is not guaranteed or endorsed by the publisher.
